# Relevance of Preoperative Cognitive Impairment for Predicting Postoperative Delirium in Surgical Medicine: A Prospective Cohort Study

**DOI:** 10.3390/geriatrics9060155

**Published:** 2024-12-06

**Authors:** Henriette Louise Moellmann, Eman Alhammadi, Philipp Olbrich, Helmut Frohnhofen

**Affiliations:** 1 Cranio-and-Maxillo Facial Surgery, University Hospital Düsseldorf, Moorenstraße 5, 40225 Düsseldorf, Germany; eman.alhammadi@med.uni-duesseldorf.de; 2 Dubai Health, Dubai P.O. Box 1853, United Arab Emirates; 3 Medical Faculty, Heinrich-Heine-Universität Düsseldorf, Universitätsstrasse 1, 40225 Düsseldorf, Germany; 4 Orthopedics and Trauma Surgery, University Hospital Düsseldorf, Moorenstraße 5, 40225 Düsseldorf, Germany; h.frohnhofen@med.uni-duesseldorf.de

**Keywords:** postoperative delirium, surgery, cognitive impairment, geriatric assessment

## Abstract

Background: Post-operative delirium is a dreaded complication after surgery in older patients. The identification of risk factors for delirium and comprehensive geriatric assessment is an extensive part of recent research. However, the preoperative assessment of risk factors, such as impaired cognition, is frequently not standardized. Methods: A comprehensive preoperative assessment was performed in 421 surgical patients to investigate the impact of preoperative cognitive impairment (PCI) on the risk of delirium and to evaluate appropriate screening tools (Six-item screener (SIS) and clock-drawing test (CDT)). Results: Both screening tools showed a significantly increased risk of delirium with *p* < 0.001 (OR 12.5, 95% [6.42; 24.4]) in SIS and *p* = 0.042 (OR 2.02, 95%CI [1.02; 4.03]) in CDT for existing cognitive impairment. A higher level of care (*p* < 0.001) and statutory care (*p* < 0.001, OR 5.42, 95%CI [2.34; 12.6]) also proved to be significant risk factors. The ROC curves of the two tests show AUC values of 0.741 (SIS) and 0.630 (CDT). The COP values for the SIS are 4 points with a Youden index of 0.447; for the CDT, the COP is 2 (Youden index = 0.177). Conclusions: The recording of PCI should be a central component of the preoperative geriatric assessment. The tools used are simple yet effective and can be easily implemented in routine clinical practice. By reliably identifying patients at risk, the available resources can be personalized and used in a targeted approach.

## 1. Introduction

Postoperative delirium (POD) is a common complication in older surgical patients, accounting for 15–25% in elective surgery [1,2] and 50–60% in emergency surgery [3,4,5,6]. Mechanisms, such as neuroinflammation, neurotransmitter imbalance, altered biological rhythms, altered brain metabolism or impaired neuronal network connectivity, can contribute to the development of delirium [7,8,9,10]. Anesthesia has a particular influence here, as the central nervous system (CNS) is the target organ of almost all anesthetics and analgesics. Since CNS dysfunction plays a central role in perioperative neurocognitive disorder [11,12], it is essential to assess the CNS before its function is dramatically altered by anesthetics [13]. In addition, other age-related comorbidities such as hypertension, diabetes mellitus and obstructive sleep apnea [14] can also increase the risk of cognitive impairment [13].

If delirium occurs, the rate of postoperative complications is increased, the length of stay in hospital is prolonged, transfer to care facilities is more frequent, the ability to cope with daily tasks is reduced, the cognitive state is reduced, the pace of long-term cognitive decline is faster, the rate of dementia and mortality is increased [15,16,17,18,19,20,21].

Preoperative cognitive impairment (PCI) is a relevant risk factor for suffering delirium. Cognitive impairments are frequently not recorded as standard within preoperative setting [17]. The medical team’s lack of understanding of the variety of delirium presentations, its erratic character, and the difficulty in assessing patients with cognitive impairment are common problems in delirium diagnosis. Clinicians typically rely on broad observational diagnosis rather than a score-based or systematic assessment for delirium. This approach may result in more patients receiving incorrect diagnoses [22,23,24,25,26]. Even though, that the awareness of delirium has increased in recent years due to announcements by various medical societies and the publication of evidence-based international guidelines [27,28,29]. However, standardized monitoring is highly challenging as preoperative cognitive impairment encompasses a broad spectrum of neurocognitive changes. Mild cognitive impairment through dementia is possible. Furthermore, deterioration can occur in memory, language, visuospatial abilities, executive functions and calculation [30,31]. The cause of PCI is complex and results from several aspects (patient- and therapy-specific) [30]. Postoperative delirium is an acute and fluctuating disturbance of attention and consciousness associated with cognitive dysfunction. It is a very common, serious, and potentially fatal disorder related to neuroinflammation and should be considered as an acute end-organ dysfunction [27,31,32]. Due to the lack of comprehensive routine diagnostics, delirium in older patients is oftentimes underestimated and not recognized. In fact, 30–40% of cases are potentially preventable [33].

Preoperative identification of predisposing factors, such as neurocognitive impairment [13,34,35] and postoperative screening for the presence of delirium, are crucial. This prospective clinical study investigates the predictive power of preoperative cognitive impairment for the occurrence of postoperative delirium in surgery.

## 2. Materials and Methods

This study included patients (over 70 years of age) who underwent surgery under general anesthesia between August 2022 and August 2023 in the departments of oral and maxillofacial plastic surgery, vascular surgery, orthopedics, trauma surgery, and general surgery. The study was approved by the Ethics Committee of the Medical Faculty of Heinrich Heine University Düsseldorf (study no.: 2022-1810) and registered in a publicly accessible database according to DvH2013, § 35 (German Register for Clinical Studies, DRKS-ID: DRKS00028614). Informed consent was obtained from all patients. The aim was to recruit at least 100 patients per discipline within one year, i.e., over 400 patients in total. All patients were recruited on presentation at the outpatient clinics or via the emergency department prior to their inpatient admission.

A comprehensive geriatric assessment was carried out as part of this study. Preoperative parameters were recorded to assess the patient’s state of health and individual risk factors. These included age, gender and body mass index (BMI) as basic demographic parameters, as well as Care level, statutory care, ACB-Score and ASA-Classification. Salahudeen MS et al. (2015) completed and refined the ACB measure, which again rates drugs on a scale of 1 to 3 [36]. Higher scores likewise correspond to a higher cognitive risk and anticholinergic burden. Salahudeen and Nishtala et al. (2016) introduced the anticholinergic burden classification (ACB) that same year. It was created in response to the requirement for a uniform technique to evaluate the anticholinergic burden of drugs. Comprehensive research on the anticholinergic effects of different medications and their possible impacts on cognitive function served as the foundation for the development of the ACB. These are ranked from 0 (no anticholinergic effects or negligible anticholinergic burden) to 3 (high anticholinergic burden with prominent anticholinergic effects) based on their potential for anticholinergic effects [36,37]. A German translation and adaptation have been made by Kiesel et al. (2018) [38]. The Six-item screener (SIS) [39] and the clock-drawing test (CDT) [40] were used to record the preoperative cognitive status. The screening tools were selected on the basis of the tools applied in the clinic (SIS and CDT). The medical staff is trained and very experienced in carrying out the tests, which increases the quality of the evaluation. The SIS is a simple and easy-to-use screening tool. It includes three questions for temporal orientation (day, month and year) and three questions for recall [39]. With a cut-off of three or more errors, the sensitivity and specificity of the six-point screener for the diagnosis of dementia was 88.7 and 88.0 [39]. As the score is the sum of the correct answers, this test can be used by anyone and easily integrated into the routine clinical care of older patients [41]. With 3 or more errors, i.e., a score of ≤3, cognitive impairment is likely [39]. The CDT is frequently used as a screening instrument to assess cognitive congestion [42,43,44]. Shulman’s method evaluates the clock drawing based on the presence and correctness of the clock circle, the numbers, the clock hands and the spacing between the numbers. Each aspect is scored 0 or 1, resulting in a total score of 0 to 6. Higher scores indicate better cognitive performance [45]. The CDT is considered an ideal cognitive screening instrument because it is fast to apply, accepted by patients, easy to score, relatively independent of culture, language and education, has good inter-rater and test–retest reliability, high sensitivity and specificity, correlates with severity and other dementia rating scores, and has predictive validity [40,41,44,46,47,48]. Cognitive abilities such as comprehension, planning, visual memory and reconstruction, visuospatial skills, motor programming and execution, numerical knowledge, abstract thinking, inhibition of drawing by perceptual features, concentration and frustration tolerance are mapped in the CDT [40,49,50,51].

Following surgery, patients were routinely screened for the presence of delirium using the NuDesc (Nursing Delirium Screening Scale), CAM (Confusion Assessment Method), CAM-ICU (Confusion Assessment Method in the Intensive Care Unit), and the 4AT.

### Statistical Analysis

Excel was used to record the data that was gathered. Jamovi (version 2.3.28 [computer software], obtained from https://www.jamovi.org, accessed on 21 September 2024) was used to perform statistical analysis. *p*-values less than 0.05 were used to determine statistical significance in each analysis. Descriptive statistics and exploratory data analysis were employed for the data’s descriptive analysis and feature inspection. When significant outliers were eliminated using boxplots, the dependent variable’s normal distribution was assessed using the Shapiro–Wilk test, and homoscedasticity was verified using Levene’s test. Mean differences between patients with or without delirium were then examined using independent *t*-tests (t). For non-normal dependent variable data, the Mann–Whitney U test (U) was used to examine mean differences. If normal distribution and variance homogeneity are violated, the robust Yuen’s *t*-test was used. A contingency table was made for categorical variables. Using the chi-square test, relationships between category variables were examined. It shows the likelihood that the study’s observations can be applied to the general population. Cramer’s V (measure of the correlation between two nominally scaled variables) was used as a measure of the effect size of the χ^2^ test (chi-square test). When interpreting Cramér’s V according to Cohen (1988), a small effect is present at V = 0.1, a medium effect at V = 0.3 and a large effect at V = 0.5 [52,53]. Binomial logistic regression analysis was used and Odd’s ratios (OR) were calculated to identify influencing factors that were considered statistically relevant at a significance level of *p* = 0.05. Significant was defined as a *p*-value of less than 0.05, very significant as a value of less than 0.01, and highly significant as a value less than 0.001. Cut-off points (COPs) were determined using ROC curves to analyze the ability to classify patients with or without delirium; we chose the COP with the highest Youden index. To determine the COPs for the two tests (SIS and CDT), the ROC curves, the areas under the ROC curves (AUC), the confidence intervals (95% CI) and the sensitivity (SE), specificity (SP), positive predictive value (PPV) and negative predictive value (NPV) were determined for each COP. We selected the COP with the highest Youden index (Youden index: SE + SP-1).

## 3. Results

The analysis included 421 patients who underwent surgery under general anesthesia in the departments of Orthopedics and Trauma Surgery, Vascular Surgery, General Surgery and Oral and Maxillofacial Plastic Surgery from August 2021–October 2023. The cohort consisted of 199 women (80.8 ± 6.7 years, 164 ± 13.7 cm, 68.0 ± 15.2 kg, 24.4 ± 5.46 kg/m^2^) and 222 men (78.8 ± 6.2 years, 173.0 ±9.25 cm, 78.5 ± 14.4 kg, kg/m^2^). The delirium rate of the entire cohort was 12.3% (*n* = 51). An overview of the distribution of patients across the individual specialist disciplines, the proportion of elective or emergency patients and the respective delirium rates can be found in Table 1.

**Figure 1:**
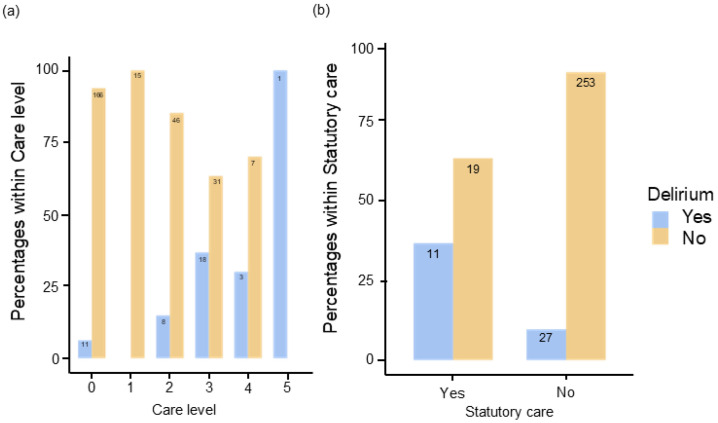
Illustration of the proportion of patients with and without delirium within (**a**) the groups of care level (0–5) and (**b**) depending on existing statutory care.

The level of care and the respective delirium rate were also recorded. The delirium rate was 5.64% (n = 11) for no or low care level (n = 195, 62.9%), 25.5% (n = 26) for moderate care level (n = 104, 33.5%) and 36.4% (n = 4) for high care level (n = 11, 3.5%). A total of 30 patients (9.6%) were under statutory care, 283 patients (90.4%) were not under statutory care. Delirium rates here were 36.7% (n = 11) and 9.6% (n = 27), respectively. The correlation between level of care and delirium was significant with χ^2^(5) = 42.1 *p* < 0.001, Cramer’s V = 0.371, but due to the small number in some groups and limited evaluation (see Figure 1a). The statistical correlation between statutory care and the occurrence of delirium was also examined using a chi-square test. With χ^2^(1) = 18.4 *p* < 0.001, Cramer’s V = 0.244, there is a purely significant correlation here. The risk of delirium was increased 5.42-fold (OR 5.42, 95%CI [2.34; 12.6]) in the presence of statutory care (see Figure 1b).

**Figure 2:**
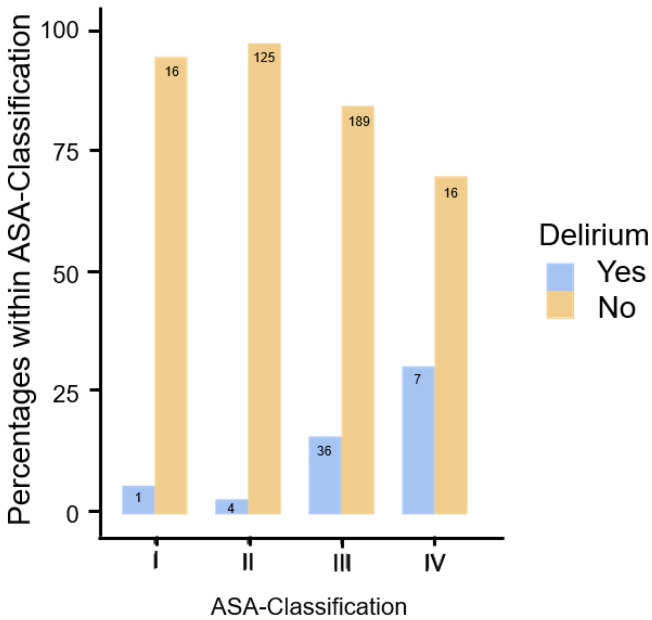
Presentation of the proportion of patients with and without delirium within the levels of the ASA classification (I–IV).

Anticholinergic burden was increased in 43 patients (10.4%) who had three or more points. There was no elevated anticholinergic burden in 370 patients. Patients with a higher anticholinergic load had a delirium rate of 25.6% (n = 11), whereas the rate for other patients is 11.0% (n = 40). The data showed a significant association (χ^2^(1) = 7.52, *p* = 0.006, Cramer’s V = 0.136). Delirium is 2.79 times more likely (OR 2.79 [95% CI: 1.31; 5.97]). To assess the general condition of the patients, the ASA classification (n = 399) and the respective delirium rate were evaluated. A total of 17 patients (4.3%) were classified as ASA I, the delirium rate was 9.1% (n = 1). An ASA II classification was present in 131 patients (32.8%), with a delirium rate of 2.9% (n = 1). In ASA III (n= 227, 56.9%) and ASA IV (n= 24, 6.0%), the delirium rate was 14.3% (n = 6) and 0.0%, respectively. With χ^2^(3) = 20.8 *p* < 0.001, Cramer’s V = 0.230, there is a significant correlation between ASA classification and delirium. Here, too, only a limited assessment is possible due to the small number of patients in some groups (see Figure 2).

The ROC curves of the two tests showed AUC values of 0.741 (SIS) and 0.630 (CDT). The COP values for the SIS are 4 points with an SE of 91.76% and a Youden index of 0.447. For the CDT, the COP is 2 (SE = 71.05%, Youden index = 0.177). This matched the cut-offs set in our descriptive analysis and in the calculation of the static correlation of the individual parameters with the occurrence of delirium (see Table 2).

The Six-item screener was performed in 408 of the total of 421 patients. On average, the patients achieved a score of 4.99 ± 1.50 points (IQR 1.00, 95%CI [4.84; 5.13]). In 14.0% of cases (n = 57), the test indicated a cognitive impairment. The CDT was performed in 335 patients. The patients scored 2.19 ± 144 points (IQR 2.00, 95%CI [2.03; 2.34]). A total of 30.5% (n = 103) patients showed evidence of cognitive impairment.

**Figure 3:**
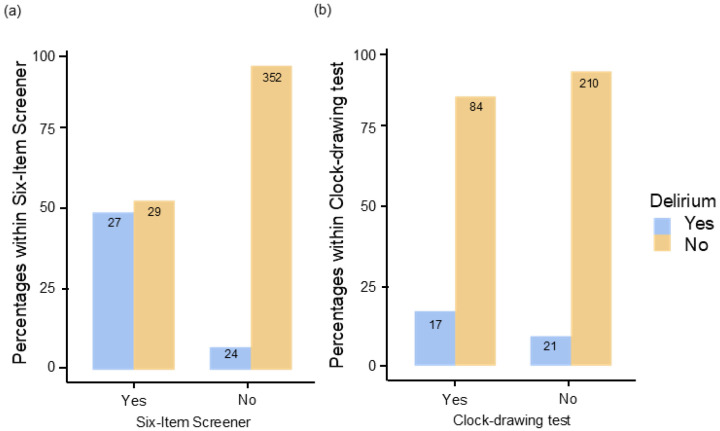
Percentage of patients with and without delirium with a (**a**) positive Six-item screener or (**b**) positive clock-drawing test.

A chi-square test was used to test for a statistical correlation between cognition and delirium. The Six-item screener showed a statistically significant correlation with χ^2^(1) = 74.4, *p* < 0.001, Cramer’s V = 0.430. The risk of experiencing delirium was increased 12.5-fold (OR 12.5, 95% [6.42; 24.4]) (see Figure 3a). The delirium rate here was 48.2% (n = 27). The CDT also shows a significant correlation with χ^2^(1) = 4.15 *p* = 0.042, Cramer’s V = 0.112. With an abnormal CDT, the risk of suffering delirium (16.8%, n = 17) is increased 2.02-fold (OR 2.02, 95%CI [1.02; 4.03]). The CDT also showed a significant correlation with χ^2^(1) = 4.15 *p* = 0.042, Cramer’s V = 0.112. With an abnormal CDT, the risk of suffering delirium is increased 2.02-fold (OR 2.02, 95%CI [1.02; 4.03]) (see Figure 3b).

If delirium was present in the patients, the scores in the preoperative Six-item screener were 3.39 ± 2.23 (IQR 3.50, 95%CI [2.77; 4.02]). If there was no delirium, they were 5.22 ± 1.20 (IQR 1.00, 95%CI [5.09; 5.34]). A total of 12.66% of patients (n = 51/403) showed signs of cognitive impairment. The delirious patients showed values of 2.95 ± 1.90 in the preoperative CDT (IQR 4.00, 95%CI [2.32; 3.57]). Non-delirious patients showed preoperative values of2.09 ± 1.34 (IQR 2.00, 95%CI [1.93; 2.24]). Cognitive impairment was probable in 11.45% (n = 38/332).

**Figure 4:**
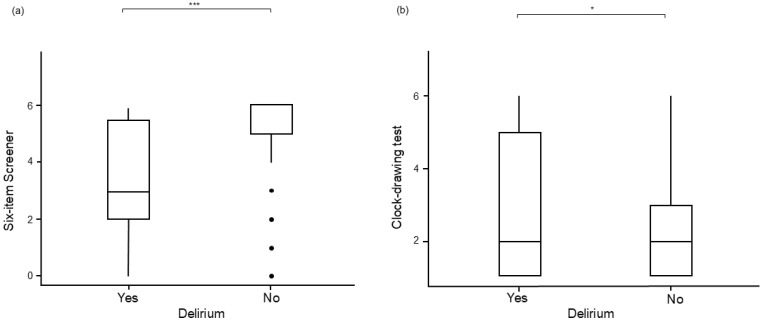
Comparison of the obtained values of (**a**) Six-item screener, and (**b**) clock-drawing test in relation to the incidence of delirium in emergency surgery (* *p* < 0.05; *** *p* < 0.001).

In the comparison of the two groups delirium vs. no delirium, significant differences were found in the Yuen’s t-test with Ty (30.4) = 3.71, *p*< 0.001, ξ^2^ = 0.687 in the Six-item screener (see Figure 4a) and with Ty (24.8) = 2.18, *p* = 0.039, ξ^2^ = 0.356 in the CDT (see Figure 4b).

A binomial logistic regression was performed to investigate the influence of age, statutory care, SIS and CDT on delirium. The binomial logistic regression model was statistically significant, χ^2^ (4) = 24.1, *p* < 0.001, resulting in a low proportion of explained variance (Backhaus et al., 2006), as shown by Nagelkerke’s R^2^ = 0.191. The overall percentage accuracy of the classification was 90.2%, with a sensitivity of 99.1% (AUC = 0.743) for delirium and a specificity of 15.2%. With a coefficient of determination of R^2^ = 0.191, a sample size of 421 and a significance level of α = 0.05, we would have a statistical power of 1-β = 1 with 4 predictors. The statistical power indicates the probability of committing a second type of error. Here, the probability of making a second type of error would be 0%. In 0% of cases, the test would not indicate significance, even if it were actually significant [54]. Of the four variables included in the regression model, one contributed significantly to the prediction of delirium: SIS (*p* = 0.026), while the other variables showed no significant effect: CDT (*p* = 0.124), age (*p* = 0.127), and statutory care (*p* = 0.680). High scores on the SIS (few errors) were associated with a reduced likelihood of experiencing delirium, OR = 0.695 (95% CI [0.504; 0.958]). All model coefficients and odds ratios can be found in Table 3.

## 4. Discussion

This prospective clinical study with 421 surgical patients over 70 years found a strong association between preoperative cognitive impairment and the risk of postoperative delirium. Depending on the level of care, patients’ ability to carry out activities of their daily lives (e.g., personal hygiene, preparing meals, taking medication, physical mobility, handling money, leisure activities, social contacts) is reduced. Furthermore, they are sometimes dependent on the help of others [55]. The need for care is associated with age, state of health, functional disability, dementia, and frailty [56]. In a cross-sectional study by Doroszkiewicz et al. (2022), 200 older people who were hospitalized in a geriatric ward were examined. Amongst others, the authors analyzed the need for care, socio-demographic parameters, cognitive functional status, and functional status (according to the Barthel scale and the I-ADL), and revealed that the degree of care dependency correlated statistically significantly with the cognitive status of the participants, *p* = 0.0001 [57]. In this research, the need for care and the presence of legal guardianship is a surrogate marker for existing cognitive impairment, which is closely related with an increased risk of suffering delirium.

Many studies revealed that anticholinergic burden increases the risk of delirium [58,59,60,61]. Other research showed no correlation between anticholinergic exposure and delirium [62,63,64,65]. In addition to the selection of the patient cohort, the choice of screening instrument also seems to explain the contradictory results. It is crucial to note that the use of anticholinergic medication probably varies between different populations, which is reflected in the development of scales for anticholinergic medication [66]. Moreover, regularly updating of drug scales is vital to capture new drugs with anticholinergic effects [66]. Additional criticism lies in the simplification of complex pharmacological issues. The number of drugs is increased without considering the linear anticholinergic effect [67]. The simple structure of the tests allows for rapid performance, but does not cover any patient-specific factors (e.g., pharmacodynamics, cholinergic reserve, endogenous anticholinergic activity) [67]. Even if the connection between delirium and anticholinergic load is controversial, we recommend evaluation as part of a preoperative assessment. Furthermore, a critical review of medication increases awareness of possible polypharmacy among older patients.

In addition to the assessment of patients’ general disease and health status, the ASA classification is one of the most valuable methods for preoperative determination of surgical and anesthesiologic risk [68]. It is frequently used to predict perioperative risk and mortality [69,70]. With a high ASA classification, the occurrence of is more likely [71] and postoperative mortality increases [68]. Furthermore, its suits as an independent risk factor for postoperative delirium in older adult patients with hip fractures [72]. The study at hand identified a higher delirium rate with increasing ASA classification, although only assessed to a limited extent. In addition to the evaluation of cognitive status, the ASA classification should therefore be used to identify patients at risk of delirium.

Using preoperative assessment (including SIS and CDT), this research demonstrates a prevalence of cognitive impairment (14–30.7%), which is in line with the recent literature [73,74,75,76,77,78,79]. In the general population, the prevalence is 5–25%, in older surgical patients up to 44% [73,74,75,76,77,78,79]. Besides the patient selection (emergency vs. elective, cardiac vs. non-cardiac), the choice of the screening tool is also relevant for the different prevalence figures. Using Mini-Cog (score ≤ 2), cognitive impairment was found to be 21% (n = 279) in an analysis of 1338 non-cardiac surgical patients (77 ± 6 years) by Weiss et al. (2023) [17]. Delirium occurred in 15% (199/1338) of patients, which is similar to the identified overall delirium rate of 12.3%. Weiss et al. (2023) showed a delirium rate of 30% among patients with cognitive impairment. The risk of delirium with a positive Mini-Cog was increased 3.3-fold with an OR of 3.3 95%CI [2.3; 4.7] [17]. This prospective clinical study was able to demonstrate similar values for the CDT with a 2.02-fold increased risk. For the SIS, the 12.5-fold increased risk is significantly higher than the reported values. A comparison of the delirium rates shows that the delirium rate is 30.0% for a positive Mini-Cog [17], 16.8% for a positive CDT and 48.2% for a positive SIS. The choice of a suitable screening tool appears to be crucial. It must be suitable for routine use in the fast-paced environment of the emergency department, as well as in the elective situation. In short, sensitive and easy-to-remember tests should be available that can be integrated into the routine history and examination without taking significantly more time to examine the patient [41].

The SIS fulfills these requirements with a sensitivity of 63% (95% CI [53%; 72%]) and a specificity of 81% (95% CI [75%; 85%]). [41]. The CDT is also easy to perform and has a high degree of sensitivity and specificity as well as concurrent and predictive validity [40]. Despite the favorable results, there are limitations to both tests. The SIS only measures two cognitive areas (temporal orientation and memory). However, if there are cognitive impairments in other areas, these are not tested and should be mapped using other tests [41]. Similarly, the CDT cannot be used to infer a specific etiology from impaired cognition or to rule out a disease if the result is unremarkable. When used as an initial screening or as a follow-up instrument, the limitations should be considered. Sejunaite et al. (2023) recommend using itemized scoring for the first screening in the case of mild cognitive deficits or in people who have not previously been examined. In addition to the pure score, the types of errors should also be documented in the evaluation in order to reflect the severity of the cognitive decline [80]. The comprehensive geriatric assessment (CGA) recommends using the SIS as an initial screening and performing the CDT if the result of the SIS is abnormal. The use of a targeted preoperative assessment can reduce the risk of postoperative delirium. Patients at risk can be recognized more quickly through the identification of risk factors and directed to perioperative management [81,82].

The cut-offs for SIS and CDT of this evaluation are comparable to those reflected in the current literature [39,40,41,46]. The definition of a cut-off ensures that patients at risk can be specifically identified, as it is not possible to provide comprehensive care to every patient across the board. However, when testing patients reveals a significantly increased risk of developing perioperative delirium, perioperative care should include a significant risk minimization [27,83]. Intensive non-pharmacological prophylaxis are advisable [84,85,86] when there are no causal drug therapy options available. After all, delirium is avoidable in up to 40–50% of cases by taking suitable preventive measures [87]. Attention must be focused entirely on excluding known delirogenic facts. Furthermore, even patients with negative tests should not be neglected in post-operative care.

Limitations are also evident in the prospective design. It is a random sample, so that selection bias cannot be ruled out. However, the incidence of postoperative delirium is consistent with the current literature. Many of the eligible patients refused to participate, which could also lead to a bias. In this case, a possible existing cognitive impairment is a reason for refusing to participate. The omission of potentially impaired patients may compromise the sensitivity of the SIS. This may have been the case when patients with more severe impairments were not included. However, as the rate of cognitively impaired patients is consistent with the extant literature, there is no evidence of bias in the inclusion of patients with more severe impairments. Furthermore, when diagnosing delirium, an underdiagnosis might occur. Patients are screened for the presence of delirium every 8 h in the ICU and twice a day in the normal ward for the first seven days after surgery. The European Society of Anesthesiology and Intensive Care (ESAIC) recommends close screening 3 times a day in the first three to five postoperative days [27]. As with the situation in this study, this cannot be applied universally.

The findings underline the relevance of routine preoperative individual assessment of cognitive status in surgical patients, which is also recommended by several professional societies [27,88]. The use of relatively simple and brief tests might aid in identifying patients at risk of delirium. Further studies should be conducted to develop preventive and therapeutic measures for at-risk patients to improve their outcome.

One of the most well-studied and successful initiatives for lowering the incidence of postoperative delirium is the Hospital Elder Life Program (HELP) [87,89,90,91]. With an emphasis on prevention and early diagnosis of postoperative delirium through the use of skilled personnel and established protocols, HELP is a multi-layer targeted intervention. A possible approach has been examined as part of the EASE initiative (Elder-Friendly Approaches to the Surgical Environment). An evidence-based, senior-friendly surgical environment with geriatric assessment was created. In this setting, the number of complications was reduced, length of stay was shortened, discharge to care facilities was reduced and mortality was reduced by 19%. The results show a reduction in complications, length of stay and discharge to care facilities [92]. These very promising and encouraging results should be investigated in further studies and projects.

## 5. Conclusions

The recording of PCI should be a central part of the preoperative geriatric assessment. This can be used in the consent and decision-making process for patients, as well as aid in the early identification of the most vulnerable individuals. The tools used are simple but effective and can be easily introduced into routine clinical practice. By reliably identifying patients at risk, available resources can be personalized and targeted. Which supportive perioperative measures are initiated for high-risk patients should be investigated in further studies.

## Figures and Tables

**Table 1:** Depiction of the various surgical specialties, the number of elective or emergency patients and the delirium rate.

Surgical Discipline	Elective/ Emergency	n	% of Total	Delirium	n	%
Orthopedic and	elective	46	11.5 %	No	40	90.9 %
Trauma Surgery				Yes	4	**9.09 %**
	emergency	111	27.7 %	No	90	81.81 %
				Yes	20	**18.18%**
Oral and Maxillofacial Surgery	elective	83	20.7 %	No	77	92,77 %
				Yes	6	**7.22 %**
	emergency	7	1.7 %	No	5	71.43 %
				Yes	2	**28.57 %**
Vascular Surgery	elective	111	27.7 %	No	102	91.89 %
				Yes	9	**8.11 %**
	emergency	16	4.0 %	No	12	75.00 %
				Yes	4	**25.55 %**
General Surgery	elective	27	6.7 %	No	26	0.0 %
				Yes	6	**7.22 %**
	emergency	0	0.0 %	No	0	0.0 %
				Yes	**0**	**0.0 %**

**Table 2:** Depiction of sensitivity (SE), specificity (SP), positive predictive value (PPV) and negative predictive value (NPV) were determined and Youden indices for each COP in the (a) SIS and (b) CDT.

**(a) Six-Item Screener**							
**Cutpoint**	**Sensitivity (%)**	**Specificity (%)**	**PPV (%)**	**NPV (%)**	**Youden’s Index**	**AUC**	**Metric Score**
0	100%	0%	87.34%	NaN%	0.000	0.741	0.000
1	98.01%	21.57%	89.61%	61.11%	0.196	0.741	0.196
2	97.44%	21.57%	89.56%	55%	0.190	0.741	0.190
3	96.59%	31.37%	90.67%	57.14%	0.280	0.741	0.280
4	91.76%	52.94%	93.08%	48.21%	0.447	0.741	0.447
5	83.81%	58.82%	93.35%	34.48%	0.426	0.741	0.426
6	54.26%	74.51%	93.63%	19.1%	0.288	0.741	0.288
**(b) Clock-Drawing-Test**							
**Cutpoint**	**Sensitivity (%)**	**Specificity (%)**	**PPV (%)**	**NPV (%)**	**Youden’s Index**	**AUC**	**Metric Score**
1	100%	0%	11.45%	NaN%	0.000	0.630	0.000
2	71.05%	46.6%	14.67%	92.57%	0.177	0.630	0.177
3	44.74%	71.77%	17%	90.95%	0.165	0.630	0.165
4	31.58%	82.99%	19.35%	90.37%	0.146	0.630	0.146
5	28.95%	92.52%	33.33%	90.97%	0.215	0.630	0.215
6	18.42%	97.62%	50%	90.25%	0.160	0.630	0.160

**Table 3:** Overview Model Coefficients: Models for predictability of the preoperative parameters (Age, statutory care, SIS, CDT) of Delirium. * Note. The cut-off value is set to 0.5.

**Model Fit Measures**							
**Model**	**Deviance**	**AIC**	**BIC**	**R²_N_**	**Overall Model Test**		
					**χ^2^**	**df**	***p***
1	141	151	169	0.191	24.1	4	< 0.001
Modelcoefficients—Delirium yes/no							
**Predictor**	**Estimate**	**SE**	**Z**	***p***	**Odds ratio**	**95% CI**	
						**lower**	**upper**
Intercept	−55.219	35.816	−1.542	0.123	0.00400	3.57 × 10^6^	4.472
CDT	0.2257	0.1466	1.539	0.124	125.317	0.940	1.670
SIS	−0.3642	0.1641	−2.219	0.026	0.69477	0.504	0.958
Age	0.0594	0.0389	1.527	0.127	106.124	0.983	1.145
Statutory care: No-Yes	−0.2969	0.7201	−0.412	0.680	0.74308	0.181	3.048

* Note. Estimates represent the log odds of “Delirium = yes” vs. “Delirium = no”.

## Data Availability

The datasets used and/or analyzed during the current study are available from the corresponding author on reasonable request.
